# Myeloid-derived suppressor cells in cancer

**DOI:** 10.1016/j.iliver.2022.06.002

**Published:** 2022-07-20

**Authors:** Jun Gao, Wei-Ren Liu, Zheng Tang, Jia Fan, Ying-Hong Shi

**Affiliations:** a Department of Liver Surgery and Transplantation, Liver Cancer Institute, Zhongshan Hospital, Fudan University, Shanghai, China; b Key Laboratory of Carcinogenesis and Cancer Invasion of Ministry of Education, Shanghai, China; c Institutes of Biomedical Sciences, Fudan University, Shanghai, China; d Shanghai Key Laboratory of Organ Transplantation, Shanghai, China; e State Key Laboratory of Genetic Engineering and Collaborative Innovation Center for Genetics and Development, School of Life Sciences, Fudan University, Shanghai, China

**Keywords:** Myeloid-derived suppressor cells, Immunosuppression, Immunostimulatory, Tumor microenvironment, Targeted therapy

## Abstract

Numerous recent studies have shown that myeloid-derived suppressor cells (MDSCs), a strongly heterogeneous population of immunosuppressive cells, are dysregulated in the presence of many cancers. MDSCs present different phenotypes and play prominent roles in the tumor microenvironment. To date, gene therapies targeting MDSCs are the most innovative and flexible methods to specifically modify the tumor microenvironment. Here, we summarize current studies related to the phenotypes, functions, and mechanisms of MDSCs and explore the therapeutic landscape of chemokines that affect the balance between subpopulations of MDSCs.

## 1 Introduction

Myeloid-derived suppressor cells (MDSCs) are a group of highly heterogeneous and immature cell populations that arise from immature myeloid cells in the bone marrow [1]. MDSCs encompass monocytes, macrophages, dendritic cells, polymorphonuclear neutrophils, and early myeloid precursors. MDSCs significantly contribute to the suppressive microenvironment of tumors and are involved in tumor-related immune responses *via* a variety of chemokines (e.g., granulocyte-macrophage colony stimulating factor [GM-CSF]) that are involved in tumor-triggered endocrine signaling to the immune system [2,3]. MDSCs extend into the peripheral lymphoid organs of patients with cancer and are a major component of tumor-infiltrating cells [4]. Tumor growth is characterized by the expansion of MDSCs, which can stimulate tumor angiogenesis, decrease the number of T cells, and inhibit T cell functions [5]. Our better understanding of these cells in murine models and in humans has allowed us to characterize their phenotypes in more detail.

Recently, the mechanisms underlying the roles of MDSCs in tumors have been explored in depth, yielding discoveries that suggest their crosstalk with cancer cells is one of the key factors affecting the induction of tolerance and the fate of tumor cells. In this review, we describe the biology of MDSCs, the mechanisms of their function, factors participating in their production, their roles in tumor immunology, and novel therapeutic approaches that target MDSCs alone or in combination with other therapies.

## 2 Biological dimension of MDSCs

MDSCs are a highly heterogeneous cell class that encompasses both immature myeloid cells and myeloid precursor cells [6]. Murine MDSCs have been shown to suppress immune reactions both *in vitro* and *in vivo*. However, for humans, only limited data are available owing to the absence of characteristic markers.

MDSCs significantly proliferate not only in lymphoid organs but also in parenchymal organs such as the liver and lungs of tumor-bearing mice and may promote metastasis to these organs [7]. MDSCs have a high plasticity profile and can differentiate into granulocytes, macrophages, or dendritic cells [8]. In the following sections, we will provide insight into the diverse heterogeneity of distinct MDSC subpopulations and the specific mechanisms through which they mediate immunosuppression or stimulation, as well as their cross-communications with tumor cells and various immune cells.

## 3 Phenotypes

Due to the remarkable heterogeneity of this group of myelomonocytic cells, the identification and classification of MDSCs remain difficult problems. Although the phenotypic characteristics of these cells in mice are usually defined by Gr-1^+^CD11b^+^, the situation in humans is more complex, as different tumor types may involve heterogeneous myeloid cell populations with different phenotypic and immunosuppressive characteristics [9].

Murine MDSCs are specifically characterized by the co-expression of Gr-1 and CD11b and can be subtyped into two large subpopulations termed granulocytic MDSCs (g-MDSCs; CD11b^+^Ly6G^+^ Ly6C^low^) and monocytic MDSCs (m-MDSCs; CD11b^+^Ly6G^−^ Ly6C^hi^) [10]. In humans, MDSCs are usually identified as cells with a CD14^−^CD11b^+^HLA^−^DR^dim^ phenotype [11]. Additionally, CD15, CD66b, and vascular endothelial growth factor receptor (VEGFR) have also been described as the characteristic markers of human MDSCs. Unfortunately, these markers are often only found in certain subsets of MDSCs [12]. A recent study reported that a novel marker, S100A9, was observed in MDSCs and could be used to identify m-MDSCs, but on the basis of current studies, there is no precise marker that can identify MDSCs [13]. Currently, two major classes of MDSCs have been recognized in humans: g-MDSCs, which express CD33, CD15, and CD11b with rare or no expression of HLA-DR and m-MDSCs, which express CD14 with rare or no expression of HLA-DR, CD49d^+^, and low levels of CD15 [10,14]. g-MDSCs primarily use reactive oxygen species (ROS) as the mechanism of immunosuppression and require effective intercellular contact with T cells, whereas m-MDSCs do not require cellular contact and rely on elevated inducible nitric oxide synthase, arginase, and cytokine secretion for immunosuppression [15]. Recent studies have found that the induction of various MDSC subpopulations correlates with specific cytokine profiles in the inducing tumors [16]. Several subpopulations have been described in various tumors, such as CD15^+^CD11b^+^, CD14^+^CD11b^+^, and CD33^+^CD11b^+^ [[17], [18], [19], [20]]. The different MDSC subtypes found in different tumor environments in humans are summarized in Table 1.

**Table 1:** Different subtypes of MDSCs in human cancers.

Tumor type	Phenotype	Reference
Renal cell carcinoma	CD33^+^	Rodriguez et al. [21]
	CD11b^+^CD14b^−^ CD15^+^	
	CD66b^+^CD11b^+^VEGF1^+^	
Non-small cell lung cancer	CD11b^+^CD14^−^ CD15^+^CD33^+^	Lin et al. [22]
	CD33^+^CD11b^+^CD14^−^PMN^−^	Navarro-Martín et al. [23]
	CD33^+^CD11b^+^CD14^+^HLA-DR^−/low^	
	SSC^low^Lin^−^HLA-DR^−/low^CD33^+^	Passaro et al. [24]
	CD13^+^CD11b^+^CD15^+^CD14^−^	
Colon cancer	Lin^−^HLA-DR^−^CD33^+^CD11b^+^	Diaz-Montero et al. [25]
	CD14^−^ CD11b^+^CD33^+^	Shimura et al. [26]
	CD45^+^CD11b^+^CD33^+^HLA-D^/low^	Fědorová et al. [27]
	CD14^+^CD15^−^	
	CD45^+^CD11b^+^CD33^+^HLA-DR^-/low^	
	CD14^−^ CD15^+^	
Breast cancer	Lin^−^HLA-DR^-^CD33^+^CD11b^+^	Diaz-Montero et al. [25]
	CD14^+^HLA-DR^-/low^	Yuan et al. [28]
	CD33^+^HLA-DR^-/neg^CD15^+^CD11b^+^	Mundy-Bosse et al. [29]
	CD11b^+^CD15^+^	
	HLA-DR^-/low^CD15^+^	Safarzadeh et al. [30]
Prostate cancer	CD14^+^HLA-DR^-/low^	Vuk-Pavlovic et al. [31]
	CD33^+^CD11b^+^ HLA-DR^-^CD14^−^	Chi et al. [32]
	CD33^+^CD11b^+^CD15^+^	Calcinotto et al. [33]
Malignant melanoma	CD14^+^HLA-DR^-/low^CD80^+^CD86^+^	Filipazzi et al. [34]
	CD33^+^CD11b^+^ HLA-DR^-/low^CD14^+^	Filipazzi et al. [35]
	CD33^+^CD11b^+^ HLA-DR^-^CD14^−^	Jordan et al. [36]
	CD33^low^CD11b ^+^ HLA-DR^-/low^ CD14^−^ CD15^+^	Stanojevic et al. [37]
Hepatocellular carcinoma	CD14^+^HLA-DR^-/low^	Hoechst et al. [38,39]
	CD33^+^CD11b^+^HLA-DR^-/low^CD14^+^ CD15^−^	Li et al. [40]
	CD33^+^CD11b^+^ HLA-DR^-^CD14^+^	Hetta et al. [41]
	LOX-1^+^ CD15^+^	Nan et al. [42]
Pancreatic carcinoma	CD15^+^CD11b^+^	Porembka et al. [43]
	Lin^−^HLA-DR^-^CD15^+^CD33^+^ CD11b^+^	Khaled et al. [44]
	CD13^hi^ (CD14^−^ CD11b^+^ CD33^+^ CD15^+^CD16^+^HLA-DR^-^ CD66^low^)	Zhang et al. [45]
Bladder cancer	CD11b^+^HLA-DR^+^, CD11b^+^CD15^+^HLA-DR^-^	Eruslanov et al. [46]
Gastrointestinal malignancies	HLA-DR^-^CD11b^+^CD15^+^, CD33^+^HLA-DR^-/low^CD14^+^	Mundy-Bosse et al. [47]
	CD14^+^HLA-DR^-^CD11b^+^	Urakawa et al. [48]

## 4 Mechanisms of activity

**Fig. 1:**
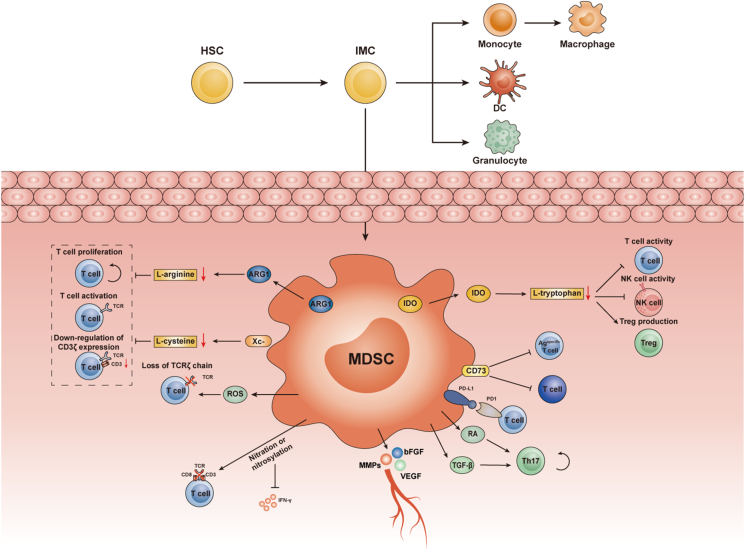
**Representative mechanisms of myeloid-derived suppressor cell (MDSC)-mediated immunosuppression.** Normally, hematopoietic stem cells (HSCs) differentiate into immature myeloid cells (IMCs), which can differentiate into granulocytes or monocytes, or further differentiate into mature macrophages or dendritic cells (DCs). In tumors, this maturation process is disrupted, resulting in a dramatic expansion of the MDSC population. (1) MDSCs expressing ARG1 and Xc-deprive T cells of L-arginine and L-cysteine to exert immunosuppressive functions *via* metabolism-related mechanisms. (2) Depletion of L-tryptophan by IDO expression, which inhibits T cell and natural killer (NK) cell activity and induces regulatory T cell (Treg) production. (3) MDSCs release reactive oxygen species (ROS), which mediate loss of the TCRζ chain. It also nitrates T cell receptor (TCR) signaling complex components and reduces the secretion of IFN-γ. (4) High CD73 expression on MDSCs drives their immunosuppressive activity. (5) Secretion of pro-angiogenic factors such as matrix metalloproteases (MMPs), vascular endothelial growth factor (VEGF), and basic fibroblast growth factor (bFGF), all of which promote angiogenesis. (6) MDSC expression of PD-L1, which targets PD-1 on T cells; and finally, retinoic acid and TGF-β secretion, which induce Th17 plasticity.

MDSCs are produced in the bone marrow and released into circulation, where they accumulate in large quantities in tumor tissues to fully exert their immunosuppressive function. Clarifying the key mechanisms through which MDSCs migrate into the tumor microenvironment could be an important means of blocking MDSCs to reverse the immunosuppressive tumor microenvironment (Fig. 1).

### 4.1 Metabolic-based mechanisms

MDSCs usually exert immunosuppressive activity in tumors, and this inhibitory effect is tightly linked to metabolic pathways including the metabolism of amino acids by arginase I and inducible nitric oxide synthase [49]. Increased nitric oxide (NO) production leads to the consumption of L-arginine, which is essential for T cells to synthesize proteins. T cells are suppressed by NO, which arrests cellular protein synthesis, decreases cellular defense against DNA damage, and blocks cell proliferation [50]. NO can also induce the nitrosylation of T cell receptors (TCRs) on tumor-infiltrating lymphocytes, and T cell migration can be impaired by chemokines in the tumor microenvironment [[51], [52], [53]]. L-cysteine is an important amino acid that affects T cell proliferation and activation [54]. MDSCs express cystine-glutamate transporters (Xc^−^), which compete with antigen presenting cells for extracellular L-cysteine uptake, thereby preventing T cells from importing L-cysteine, which in turn affects T cell function [55]. Furthermore, MDSCs can express indoleamine-2,3-dioxygenase, which consumes L-tryptophan, and the resulting metabolite kynurenine decreases the activity of T cells and natural killer (NK) cells and induces regulatory T cell (Treg) production [56,57]. g-MDSCs also suppress T cells *via* the accumulation of ROS, which stimulates T cell apoptosis and the expression of nitrate TCRs [58]. The accumulation of MDSCs has been measured in the peripheral blood of patients with glioma, and there is a possibility that this promotes T cell immunosuppression through the increased plasma levels of arginase and G-CSF [59,60].

### 4.2 Immunomodulation-related mechanisms

The specific TCR complex of CD8^+^ T cells can be nitrated by MDSCs, resulting in diminished interactions between TCRs and CD3ζ and CD8, which consequently reduces the expression of various TCR-related molecules. Correspondingly, activation, amplification, and IFN-γ secretion in response to the specific TCR antigen are also reduced [61]. MDSCs may block the function or activity of CD8^+^ T cells mediated by the interaction of PD-1 and PD-L1 *via* phagocytosis [62]. MDSCs can play an immunosuppressive role not only in Ag-specific T cell responses but also in mitogen-activated T lymphocytes [63]. The generation of adenosine by CD73, which is highly expressed on g-MDSCs, promotes their expansion and facilitates their immunosuppressive activity [64].

In addition to inhibiting CD8^+^ T cell function, MDSCs also interact with other immune cells. CD14^+^HLA-DR^−/low^ MDSCs induce Foxp3^+^ Treg cells, whereas CD14^+^HLA-DR^+^ monocytes promote the generation of IL-17-secreting RORc^+^ Th17 cells when cocultured with naive CD4^+^ T cells [65]. MDSC-derived TGF-β and retinoic acid also induce Th17 plasticity [66]. In the liver and spleen, MDSCs can significantly inhibit the cytotoxic effects of NK cells, and the specific mechanism of action may be the suppression of IFN-γ responses by NK cells through membrane binding of TGF-β1 by MDSCs [3,67]. Further analysis of the intricate relationships between MDSCs and other types of cells will help uncover new biological functions.

### 4.3 Regulation of angiogenesis

Several studies have reported that MDSCs play a key role in angiogenesis. MDSCs are observed in the vicinity of tumor blood vessels, and their pro-angiogenic effect is probably relevant to the secretion of pro-angiogenic factors such as matrix metalloproteases (MMPs), VEGF, and basic fibroblast growth factor or their capability to differentiate into endothelial cells [68,69]. Yang et al. showed that MMP9 was abundantly secreted by Gr-1^+^CD11b^+^ MDSCs and that deleting MMP9 impaired vessel density, vessel maturation, and tumor growth [70]. Interestingly, Min et al. found that MDSCs in the tumor microenvironment express large amounts of VEGFR2, which can be activated by tumor cells or themselves to produce more VEGF. Thus, the VEGF/VEGFR pathway in MDSCs may establish a positive feedback loop to maintain tumor angiogenesis [71]. The regulator of G protein signaling-2 (Rgs2) has been shown to be a crucial modulator of the pro-angiogenic functions of MDSCs in the tumor microenvironment by regulating the production of MCP-1 [72].

## 5 Regulation and differentiation

The secretion of growth factors and cytokines such as IL-6, IL-10, SCF, G-CSF, and CCL2 by primary tumors contribute to altering bone marrow myelopoiesis, which leads to MDSC expansion and mobilization [73]. Under normal conditions, MDSCs can compartmentalize into macrophages, dendritic cells, and even endothelial cells, demonstrating their advanced plasticity profile.

Although several different signaling pathways may be involved in the inhibitory pathways of MDSCs, the one involving IL4Rα/STAT6 is crucial, as it regulates TGF-β production and arginase activation and is closely related to STAT3 and STAT1 activation, enabling peroxynitrite and ROS production and MMP9 expression [74,75]. Wu et al. found that snit-inflammatory PPARγ in myeloid-lineage cells can control proinflammatory cytokine synthesis and induce MDSC activation and immunosuppression along with cancer progression. Furthermore, MDSCs inhibit wild-type CD4^+^ T cell proliferation and lymphokine production *in vitro* and *in vivo* [76]. Activated CD4^+^ T cells, which are antigen-specific, may strengthen the immunosuppressive effect of MDSCs. This phenomenon may involve a negative feedback loop that controls the dysregulated immune response in cancer [77].

Several cytokines and chemokines participate in MDSC function. MDSCs, which are attracted to tumors by CXCL5, induce epithelial–mesenchymal transition in melanoma cells *via* the EGF, HGF, and TGF-β signaling pathways [78]. TLR2 and MYD88 in MDSCs lead to the activation of MDSCs *via* increased IL-6 expression and STAT3 phosphorylation [79]. Additionally, neutrophilic granule protein has been identified as a negative regulator of MDSC-mediated metastasis [80]. TGFβ1/PTEN double conditional knockout (2cKO) mice with head and neck squamous cell carcinoma showed increased MDSC infiltration [81].

Increased numbers of MDSCs can be induced by tumor exosomes and soluble factors such as GM-CSF. Exosomes also increase MDSCs *via* TGFβ and prostaglandin E2 (PEG_2_) regulation. Additionally, the HSP72 on exosomes may affect MDSC turnover, which is regulated by activated T cell-mediated Fas-FasL apoptosis [82]. Src homology 2 domain-containing inositol 5′-phosphatase-1 may facilitate the expansion and function of MDSCs, thereby regulating the tumor immune microenvironment and contributing to the progression of pancreatic tumors [83]. Mice lacking hemopoietic expression of 5-lipoxygenase (5LO) exhibit a decreased expansion of MDSCs in the mesenteric lymph nodes, spleen, and primary cancer site [84]. PEG_2_ plays a prominent role in the MDSC generation triggered by CXCL12/CXCR4 signaling [85,86]. PEG_2_ is essential for the development of MDSCs and enhancing the accumulation of MDSCs. Interestingly, MDSCs express high levels of COX-2 and are a major source of PEG_2_ secretion in human cancer [87,88]. The COX-2 pathway facilitates gliomagenesis by actively contributing the systemic evolution of CD11b ^+^ Ly6G^hi^Ly6C^low^ MDSCs and their accumulation in the tumor microenvironment, where cytotoxic T lymphocyte infiltration is accordingly limited [89]. Suppression of the PIR-B signaling axis induces MDSC transformation into M1-type macrophages [90].

## 6 MDSCs in cancer

### 6.1 Suppressive activity of MDSCs

MDSCs are generally thought to affect immunosuppressive pathways and thereby promote cancer growth and progression. Several experiments have shown that MDSCs play immunosuppressive roles in cancer. MDSCs are abundant in the bone marrow and peripheral circulation of patients with pancreatic adenocarcinoma and are correlated with disease stage. Likewise, murine tumors recruit MDSCs that suppress CD8^+^ T cells *in vivo*, thus contributing to cancer growth *in vivo* [91]. Relative to healthy donors, there are increased numbers of CD15^+^CD16^low^ MDSCs in patients with terminal malignant tumor. T cell proliferation is strongly inhibited by MDSCs and patients with elevated levels of CD15^+^CD16^low^ MDSCs tend to have worse prognoses [92]. An increased number of MDSCs was observed in the spleen and peripheral blood of mice treated with 4-nitroquinoline-1-oxide (4NQO), which can cause oral cancer [93]. The number of MDSCs is positively correlated with systemic CD3^+^ CD8^+^ T cells. In bladder carcinoma, there are increased numbers of MDSCs with CD14^+^HLA-DR^−/low^ compared with controls, and the level was related to sex, tumor number, tumor size, pathological grade, and clinical stage. T cell proliferation and IFN-γ production were markedly reduced in these patients, and this suppression was partially reversed by L-arginine or anti-TGFβ treatment [28]. In gastrointestinal malignancies, upregulated plasma IL-6 was found to be linked with CD33^+^HLA-DR^−^CD15^+^ MDSCs, while upregulated plasma IL-10 was linked with CD33^+^HLA-DR^−^CD15^−^ MDSCs, which are associated with worse prognosis; additionally, the percentages of CD15^+^ and CD15^−^ cells were negatively linked with IFN-α-induced STAT1 phosphorylation in CD4^+^ T cells. A higher proportion of CD33^+^HLA-DR^−/low^CD14^+^ cells is related to longer overall survival [94]. MDSC-derived S100A8/A9 interacts with RAGE and carboxylated glycans on colon cancer cells to facilitate activation of the MAPK/NF-κB axis, which promotes tumor growth and metastasis [95]. MDSCs promoted hepatocellular carcinoma development and sensitization by inhibiting TLR ligand-induced IL-12 production in dendritic cells *via* generating IL-10 and suppressing T cell stimulatory activity in dendritic cells [96]. Compared with controls, MDSCs are significantly elevated in pancreatic, esophageal, and gastric cancers; MDSC ratios, accompanied by arginase I and IL-13, are also correlated with an increased risk of death [97]. In summary, MDSCs represent one of the many possible pathways used by tumors to create an immunosuppressive environment.

### 6.2 Immune stimulation and antitumor activity: another face of MDSCs?

New evidence suggests that MDSCs can also act as an immunostimulants rather than only as immunosuppressive agents. Some studies have shown that the presence of MDSCs is not always associated with inhibited T cell function [17]. The development of immunostimulatory properties in MDSCs can occur when they are placed in an appropriate cytokine environment. Bronte et al. showed that *via* certain cytokines, MDSCs could differentiate into antigen presenting-like cells. Their study further demonstrated that in the presence of IFN-γ and TNFα- or IL-12, suppressive CD11b^+^Gr-1^+^CD31^+^ cells are converted to stimulatory cells (with CD86 upregulation), thereby enhancing the cytotoxic T lymphocyte response *in vitro* [98]. In another study, Narita et al. demonstrated that CD11b^+^Gr-1^+^ cells from tumor-bearing mice can differentiate *in vitro* into functional CD11c^+^ cells (which are capable of generating cytotoxic T lymphocytes) or F4⁄80^+^ suppressor macrophages when Th1 cytokines and tumor-derived cytokines are present in the microenvironment, which, in turn, activates immune responses [99]. Additionally, MDSCs can also affect NK cells, another important effector cell that plays a role in antitumor immunity. Several studies have shown that MDSCs inhibit the function of NK cells through different mechanisms [61,100]. However, in a study that used a RMA-S lymphoma (NK-sensitive) tumor model, Naush et al. demonstrated the activation of NK cells by CD11b^+^Gr-1^+^F4/80^+^ MDSCs. These suppressor cells exert antitumor effects by expressing RAE-1, a ligand that activates the NKG2D receptor to induce IFN-γ production by NK cells [101]. These studies suggest that although MDSCs are a class of immunosuppressive cells, they can act as immunostimulants under certain circumstances.

## 7 Therapies targeting MDSCs

**Fig. 2:**
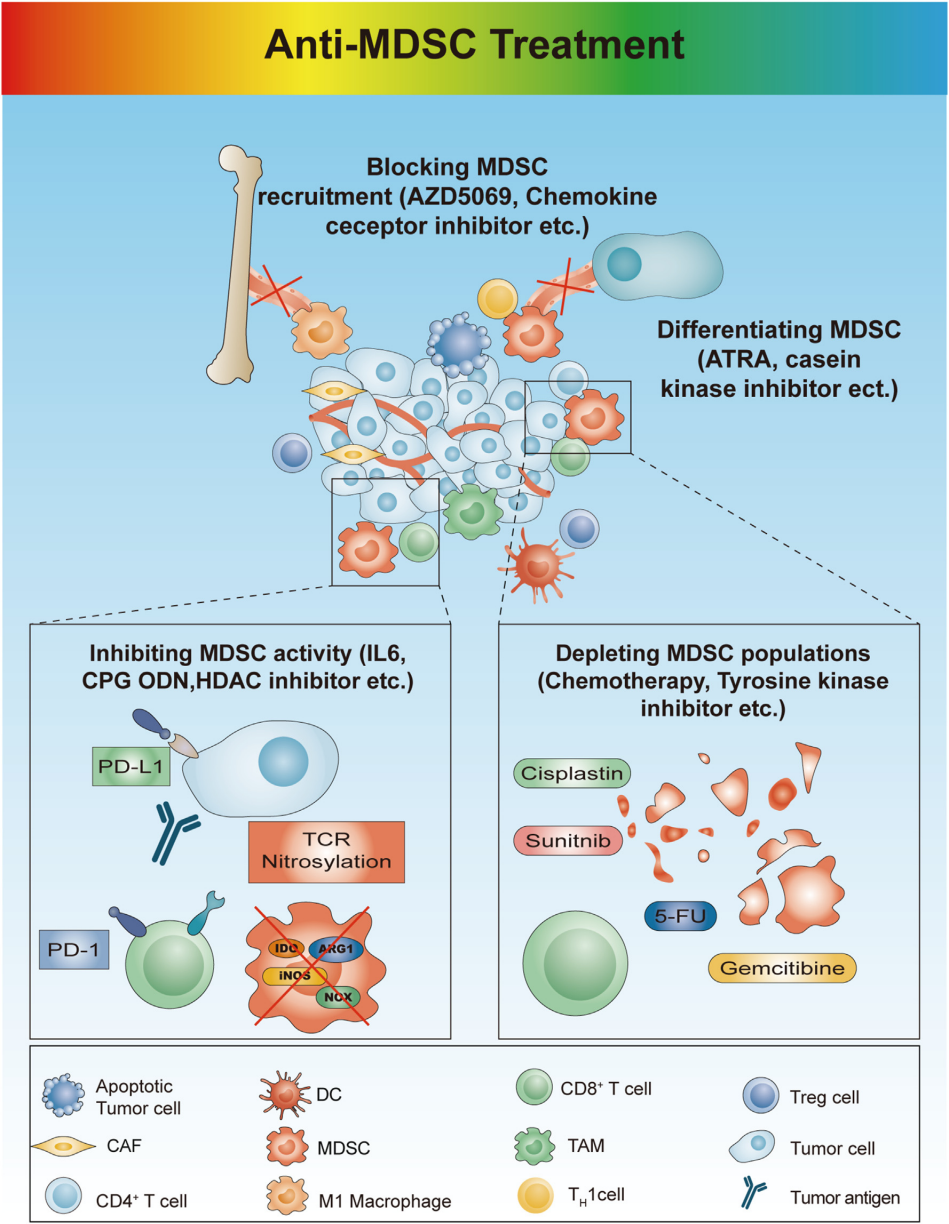
**Therapeutic approaches that target different mechanisms of myeloid-derived suppressor cells (MDSCs).** These approaches include: (1) interrupting MDSC recruitment or maturation by targeting the chemokine receptors that recruit MDSCs; (2) promoting the differentiation of MDSCs into mature, non-suppressive cells; (3) reducing reactive oxygen species (ROS) production and attenuating the immunosuppressive functions of MDSCs by downregulating the expression of ARG1 or inducible nitric oxide synthase (iNOS), and so on; (4) depleting the MDSC population by chemotherapy or tyrosine kinase inhibitors.

Various approaches of suppressing MDSCs have been investigated. To date, several strategies have been applied for MDSC-targeted therapy: (1) interrupting MDSC recruitment or maturation; (2) promoting the differentiation of MDSCs into mature, non-suppressive cells; (3) decreasing levels of MDSC infiltration; (4) functionally inhibiting MDSCs (Fig. 2). The growing understanding of MDSC populations with potent immunosuppressive properties that promote tumor development is being translated into novel therapeutic approaches. In addition to direct antitumor action, several antitumor drugs have been proven to inhibit immunosuppressive cells such as MDSCs [102]. Drugs such as gemcitabine, the tyrosine kinase inhibitor sunitinib, 5-fluorouracil (5-FU), and noncytotoxic doses of drugs, such as paclitaxel, have been shown to act on the immune system [102,103]. Recently reported targeted therapies for MDSCs in cancer are summarized in Table 2.

**Table 2:** Agents studied for their effects on MDSCs and their mechanisms.

Agent investigated	Effects on MDSCs	References
IL-6	Reduced MDSCs activity	Rebekka et al. [104]
CpG ODN	Reduced MDSCs activity	Shirota et al. [105]
AZD9150	Reduced MDSCs activity	Shastri et al. [106]
Tadalafil	Reduced MDSCs activity	Zhang et al. [107]
Sildenafil	Reduced MDSCs activity	Jiang et al. [108]
Celecoxib	Reduced MDSCs activity	Fujita et al. [109]
Entinostat	Reduced MDSCs activity	Orillion et al. [110]
Gemcitabine	Depleted MDSC	Sasso et al. [111]
Cisplatin	Depleted MDSC	Takeyama et al. [112]
5-Fluorouracil	Depleted MDSC	Pilot et al. [113]
5-Azacytidine	Depleted MDSC	Lu et al. [114]
Sunitinib	Depleted MDSC	Ko et al. [115]
Ibrutinib	Depleted MDSC	Stiff et al. [116]
Anti-IL4Rα aptamer	Depleted MDSC	Liu et al. [117]
IL-12 and CPA	Depleted MDSC	Medina-Echeverz et al. [118]
CTX and AdIL-12	Decreased MDSCs	Malvicini et al. [119]
IL-1ra	Decreased MDSCs	Triozzi et al. [120]
A plasmid encoding the HN	Decreased MDSCs	Ni et al. [121]
CTX and ATRA	Decreased MDSCs	Song et al. [122]
DC/mBORIS	Decreased MDSCs	Mkrtichyan et al. [123]
HC-Ad/RUmIL-12	Decreased MDSCs	Vergati et al. [124]
CCL2 antagonist + anti-PD-1 antibody	Decreased MDSCs recruitment	Wang et al. [125]
Maraviroc (CCR5)	Decreased MDSCs recruitment	Ban et al. [126]
AZD5069 (CXCR2)	Decreased MDSCs recruitment	O'Byrne et al. [127]
Reparixin (CXCR2)	Decreased MDSCs recruitment	Goldstein et al. [128]
SX-682 (CXCR2)	Decreased MDSCs recruitment	Greene et al. [61]
Plexidartinib (CSF-1R)	Decreased MDSCs recruitment	Yang et al. [129]
ATRA	Induced the differentiation of MDSC	Liu et al. [130]
Docetaxel	Induced the differentiation of MDSC	Chen et al. [131]
Paclitaxel	Induced the differentiation of MDSC	Kim et al. [132]
Activated T cells	Suppressed MDSCs differentiation	Thakur et al. [133]
CpG	Facilitated the maturation and differentiation of MDSCs and reduced the proportion of Ly6G^hi^ MDSCs	Zoglmeier et al. [134]
ATRA	Enhanced MDSC differentiation into mature, non-suppressive cells	Nefedova et al. [135]
Bevacizumab and EGFR TKI	Reduced the expression of circulating S100A9 positive M-MDSCs	Feng et al. [136]

## 8 Conclusions and perspectives

Recently, MDSCs have been the subject of several studies, especially regarding tumor immunology, but there remain many unanswered questions about MDSCs and their role in antitumor immunity. The main difficulty facing MDSC studies is the lack of specific markers and their heterogeneity, which make it complicated to analyze MDSCs. Furthermore, the precise conditions required to induce the stimulatory activity of MDSCs *in vivo* need to be discovered. Greater effort should be put into carefully and rigorously screening patients enrolled in MDSC trials, particularly with regards to disease characteristics and treatment history. Additionally, these studies should incorporate data on disease progression and prognosis, as well as immune responses to immunotherapy. These data are essential to uncovering the true impact of MDSCs on cancer progression and will help elucidate the roles of these mysterious cells.

Although our understanding of the mechanisms that regulate the immunosuppressive activity of MDSCs has increased rapidly in recent years, the immunosuppressive role of MDSCs has raised many questions about the balance between immune stimulation and immunosuppression in cancer immunotherapy. Without counteracting the resulting immunosuppression, antitumor immunotherapy often leads to unsatisfactory outcomes in terms of achieving objective responses and prolonging overall survival [55]. Moreover, in addition to conventional assays for monitoring immune responses in cancer immunotherapy, ideal assays would measure the balance between immune stimulation and immunosuppression.

With the constant increase of in-depth research on MDSCs, we believe that in the near future, the biological properties of MDSCs will be elucidated in depth. Effective new therapies that target human MDSCs and their subtypes will help improve the effectiveness of immunomodulatory treatments for cancer.

## Funding

This study was supported by grants from the 10.13039/501100001809National Natural Science Foundation of China (No. 81773067, 82073217, 82073218, and 82003084), Shanghai Municipal Science and Technology Major Project (No. 2018SHZDZX05), Shanghai Municipal Key Clinical Specialty, CAMS Innovation Fund for Medical Sciences (CIFMS) (2019-I2 M-5-058), and 10.13039/501100012166National Key R&D Program of China (2020YFE0202200).

## Author contributions

Writing-review and editing by J.G., W.-R.L., Z.T., J.F., and Y.-H.S.; figures designed by J.G. and W.-R.L.; supervision by Y.-H.S.

## Data Availability

No data, models, or code were generated or used during the study.
